# Unlocking the Potential of Genomic Data to Inform Typhoid Fever Control Policy: Supportive Resources for Genomic Data Generation, Analysis, and Visualization

**DOI:** 10.1093/ofid/ofad044

**Published:** 2023-06-02

**Authors:** Megan E Carey, Zoe A Dyson, Silvia Argimón, Louise Cerdeira, Corin Yeats, David Aanensen, Gerald Mboowa, Stephen Baker, Sofonias K Tessema, Anthony M Smith, Iruka N Okeke, Kathryn E Holt

**Affiliations:** Cambridge Institute of Therapeutic Immunology and Infectious Disease, Department of Medicine, University of Cambridge, Cambridge, United Kingdom; IAVI, Chelsea & Westminster Hospital, London, United Kingdom; Department of Infection Biology, Faculty of Infectious and Tropical Diseases, London School of Hygiene and Tropical Medicine, London, United Kingdom; Department of Infection Biology, Faculty of Infectious and Tropical Diseases, London School of Hygiene and Tropical Medicine, London, United Kingdom; Department of Infectious Diseases, Central Clinical School, Monash University, Melbourne, Australia; Wellcome Sanger Institute, Wellcome Genome Campus, Hinxton, United Kingdom; Centre for Genomic Pathogen Surveillance, Big Data Institute, Nuffield Department of Medicine, University of Oxford, Oxford, United Kingdom; Department of Vector Biology, Liverpool School of Tropical Medicine, Liverpool, United Kingdom; Centre for Genomic Pathogen Surveillance, Big Data Institute, Nuffield Department of Medicine, University of Oxford, Oxford, United Kingdom; Centre for Genomic Pathogen Surveillance, Big Data Institute, Nuffield Department of Medicine, University of Oxford, Oxford, United Kingdom; Africa Centres for Disease Control and Prevention, Addis Ababa, Ethiopia; Cambridge Institute of Therapeutic Immunology and Infectious Disease, Department of Medicine, University of Cambridge, Cambridge, United Kingdom; IAVI, Chelsea & Westminster Hospital, London, United Kingdom; Africa Centres for Disease Control and Prevention, Addis Ababa, Ethiopia; Division of the National Health Laboratory Service, Centre for Enteric Diseases, National Institute for Communicable Diseases, Johannesburg, South Africa; Department of Pharmaceutical Microbiology, Faculty of Pharmacy, University of Ibadan, Ibadan, Nigeria; Department of Infection Biology, Faculty of Infectious and Tropical Diseases, London School of Hygiene and Tropical Medicine, London, United Kingdom; Department of Infectious Diseases, Central Clinical School, Monash University, Melbourne, Australia

**Keywords:** analytic tools, data visualisation, genomics, *Salmonella* Typhi, typhoid fever

## Abstract

The global response to the severe acute respiratory syndrome coronavirus 2 (SARS-CoV-2) pandemic demonstrated the value of timely and open sharing of genomic data with standardized metadata to facilitate monitoring of the emergence and spread of new variants. Here, we make the case for the value of *Salmonella* Typhi (*S*. Typhi) genomic data and demonstrate the utility of freely available platforms and services that support the generation, analysis, and visualization of *S.* Typhi genomic data on the African continent and more broadly by introducing the Africa Centres for Disease Control and Prevention's Pathogen Genomics Initiative, SEQAFRICA, Typhi Pathogenwatch, TyphiNET, and the Global Typhoid Genomics Consortium.

## Teaser

We describe freely available platforms that support the generation, analysis, and visualization of *Salmonella* Typhi genomic data, highlight opportunities to access training and tools, and outline remaining challenges.

Whole genome sequencing (WGS) is a powerful tool that can improve our understanding of typhoid epidemiology and the burden of antimicrobial resistance (AMR). WGS data can provide valuable information about transmission pathways that can inform targeting of interventions and has facilitated investigations of the molecular mechanisms of new resistance phenotypes, including extensively drug-resistant (XDR) typhoid in Pakistan [1]. By providing standardized information about AMR profiles in circulating bacteria, WGS has the potential to direct local treatment guidelines and policies to extend the lifespan of existing antimicrobials. Genomic data can also inform the prioritization of vaccine introduction, and phylogenetic analysis can be used to measure the impact of vaccines on bacterial populations. Despite the value of WGS for informing typhoid control, genomic surveillance is not universally implemented, and many barriers to access exist, including cost, supply chain challenges, and lack of access to bioinformatic training [2]. Here, we seek to highlight freely available resources to support the generation, analysis, and visualization of *S.* Typhi WGS data to inform policy.

## AFRICA PATHOGEN GENOMICS INITIATIVE

In October 2020, the Africa Centres for Disease Control and Prevention (Africa CDC) launched a continent-wide pathogen genomics initiative (Africa PGI) jointly with public, philanthropic, and private sector partners with the short-term goal of accelerating severe acute respiratory syndrome coronavirus 2 (SARS-CoV-2) sequencing in Africa [3]. A longer-term ambition was to strengthen Africa's ability to use genomics for surveillance and rapid response to additional infectious disease threats, as well as facilitating of endemic disease control and elimination. Africa PGI has 4 major components:

1. *A pan-African network of genomic laboratories and bioinformatics institutes.* This includes providing national public health institutes and national reference laboratories with sequencing platforms, data systems, and quality assurance systems as well as the establishment of a continent-wide laboratory referral network.
2. *Workforce development.* The initiative will support training of laboratory technicians, bioinformaticians, and public health specialists to facilitate the generation and translation of pathogen genomic data to inform public health policy decisions.
3. *Creating enabling mechanisms to support sustainable genomics-based surveillance in the continent*. This includes developing harmonized best practices for the collection, storage, and utilization of biological specimens, managing biorepositories, and the ethical use of genomic data. In addition, the initiative will facilitate the donation of equipment, reagents, and supplies.
4. *Implementation of priority pathogen genomics use-cases.* The initiative will support the implementation of priority genomics use-cases as identified by member countries and the community of experts, who will support the sharing of best practices and affordable genomic tools, as well as setting standards and priority genomics use-cases in alignment with regional and country priorities.

These activities will contribute to the creation of a functional continent-wide pathogen genomics surveillance system, skilled workforce, and tools to help the Africa CDC and national public health institutes fulfill their mandate of reducing the burden of infectious disease and proactively counter emerging and reemerging infections.

The Africa PGI continues to strengthen public health genomics and bioinformatics capacity in Africa through financial and in-kind support from the African Union, Bill & Melinda Gates Foundation, Illumina, US Centers for Disease Control and Prevention, Rockefeller Foundation, and Oxford Nanopore Technologies and is coordinated by the Africa CDC and the African Society for Laboratory Medicine. As of September 2022, a total of 31 member states have local sequencing capacity, as compared to only 7 in 2019. During this time, 43 sequencing machines were distributed to laboratories in 29 African Union member states, and supplies, reagents, and training were provided to >400 laboratories. This expanded sequencing capacity, and the sample referral network has already contributed significantly to achieving key milestones, including >117 000 SARS-CoV-2 genomes shared via the Global Initiative on Sharing All Influenza Data (GISAID) as of September 2022, a 22-fold increase from December 2020. Of these sequences, >96% were generated by national and regional sequencing laboratories within the African continent.

In spite of these early successes, several challenges remain, including (1) scaling up genomics and bioinformatics capacity to include additional countries/regions and to augment sample volume capacity; (2) implementing standardized processes and protocols; (3) improving the supply chain for sequencing reagents and consumables; (4) consolidating the sample referral network; (5) training additional genomics and bioinformatics personnel; and (6) developing continental, regional, and national policies to enable effective implementation of public health pathogen genomics, real-time data sharing, and the integration of genomics to existing disease surveillance systems.

## SEQAFRICA

While the cost of sequencing is decreasing, many laboratories and public health institutes that could benefit from sequence-derived information cannot afford to generate it. In addition to the monetary cost of generating high-quality genomic DNA, preparing and analyzing sequencing libraries, generating sequence reads, and performing quality assurance and analysis, the dearth of expertise required to perform these tasks poses a problem in many settings on the African continent [4]. Pathogen sequencing is most easily justified by showcasing data from sequencing [5]. This creates a ratchet effect where those that require sequencing cannot justify the need and therefore cannot access the monetary and training resources to be able to generate or analyze their own sequence data.

**Figure 1.**
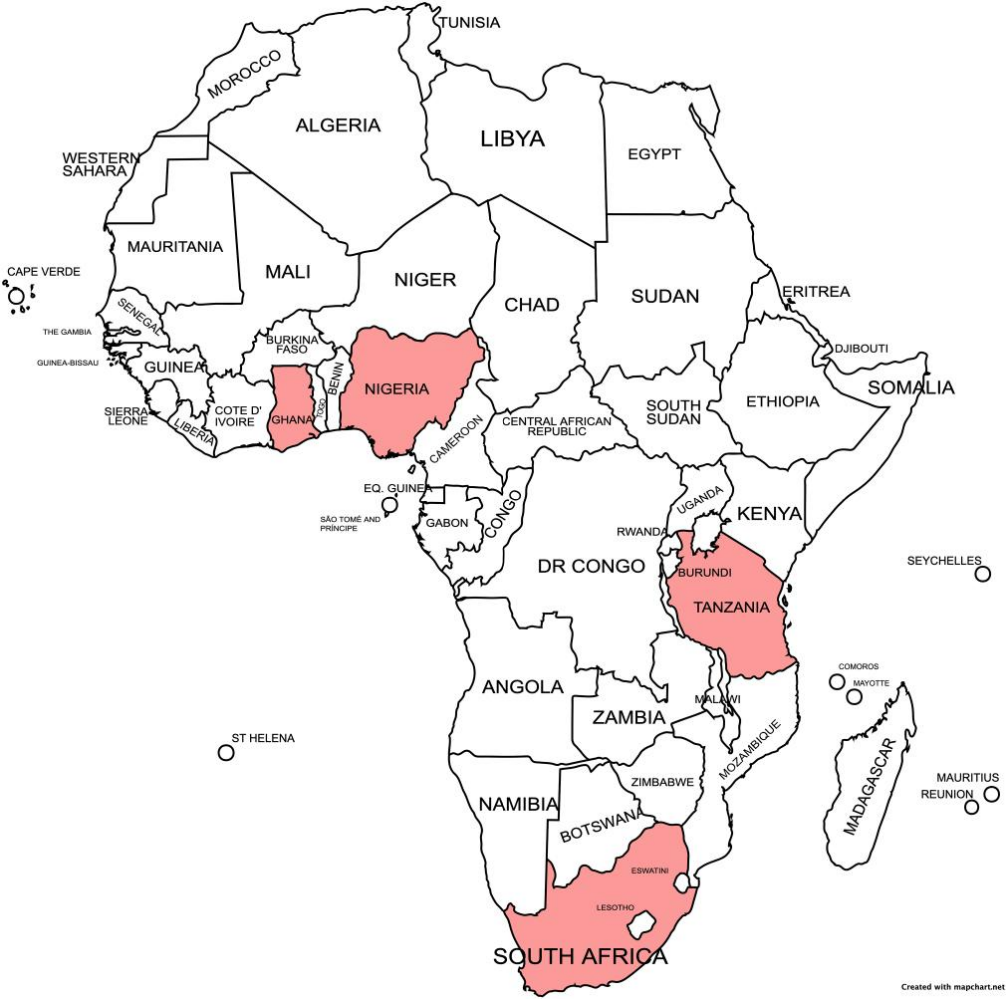
SEQAFRICA's objectives are to bring whole genome sequencing into antimicrobial resistance surveillance in Africa and to build capacity sustainably through training. The consortium has 3 regional sequencing centers, in Nigeria (University of Ibadan), Tanzania (Kilimanjaro Clinical Research Institute), and South Africa (National Institute for Communicable Diseases [NICD]); a national center in Ghana (Noguchi Memorial Institute for Medical Research); and a COVID-19 response center in South Africa (NICD Center for Respiratory Diseases and Meningitis).

Whole genome sequencing is a key tool in understanding mechanisms and transmission dynamics of AMR. Recognizing this, the Fleming Fund, which has provided support for AMR surveillance in >20 low- and middle-income countries (LMICs), has funded SEQAFRICA. The goals of SEQAFRICA are to integrate WGS into AMR surveillance on the African continent and to sustainably build capacity for generating and understanding sequence data for surveillance. The SEQAFRICA consortium (https://antimicrobialresistance.dk/seqafrica.aspx) is comprised of regional sequencing centers in Nigeria, Tanzania, and South Africa; a national sequence center in Ghana; and a South African coronavirus disease 2019 (COVID-19) response center (Figure 1). The consortium is coordinated by the Danish Technical University (DTU).

SEQAFRICA augments surveillance for pathogens of AMR importance. This includes the WHO priority organisms as well as species for which there may be local priorities. SEQAFRICA receives and reviews sequencing proposals from clients across the continent and provides free sequencing support for high-priority projects to inform AMR epidemiology. This modus operandi provides access to sequencing for high-priority initiatives that lack adequate resources and facilitates sequencing of small isolate collections of rarer organisms that can fill important surveillance gaps. To date, sequenced isolates have originated from public health institutes, African Fleming Fund Fellows leading surveillance in their respective countries, and other researchers from 15 different African countries. Completed genomes are placed in the public domain within 3 months of completion, so SEQAFRICA is rapidly filling critical surveillance and research gaps with bacterial genome data from Africa.

Established in 2018, SEQAFRICA is now more than three-quarters of the way toward achieving its goal of sequencing 16 000 genomes. *Salmonella* species, including *S.* Typhi, have been priorities from the start, although SEQAFRICA has received few *S.* Typhi sequencing requests. This lack of demand likely reflects the low isolation rate of *S.* Typhi on the continent, which is due to poor access to blood culture, as well as initial limited awareness of the program and the benefits of *S.* Typhi genome sequencing. Additionally, most *S.* Typhi isolates are obtained as part of externally funded term-limited research and surveillance projects, which often have their own arrangements for sequencing [6]. Such initiatives generate a wealth of data, but these tend to come from a smaller range of locales than routine, nationally owned sequencing, like that conducted by the National Institute for Communicable Diseases (NICD) in South Africa [7, 8]. For countries establishing surveillance, AMR and *S.* Typhi surveillance are mutually enhancing. For example, Nigeria has found that boosting AMR surveillance in a system that includes a genomic surveillance component is 1 way to expand the number and diversity of *S.* Typhi genomes [9]. For countries that do not have their own genomic AMR surveillance, SEQAFRICA represents an excellent option to generate *S.* Typhi genomic data and is therefore synergistic with Fleming Fund Country grant projects focused on constructing the architecture of national surveillance systems, including building clinical microbiology at sentinel and national levels.

Through SEQAFRICA, Yamba et al sequenced invasive *Salmonella* isolated at the University Teaching Hospital, Zambia between January 2018 and December 2019 [10]. In total, 58 of 76 (76%) *Salmonella enterica* isolates from 7180 blood cultures performed during the study period were *S.* Typhi; 46 of these were sequenced at NICD in South Africa. Genomic surveillance uncovered AMR mechanisms for >50% of the *S.* Typhi that were multidrug-resistant (MDR), revealing that the most common cluster was closely genetically related to isolates from the 2010 typhoid outbreak in Zambia. Sequencing of contemporary *S.* Typhi isolates is currently underway at SEQAFRICA.

Awareness of the value of sequencing, as well as the understanding of how to implement it, will grow with training programs, including those offered by SEQAFRICA. The consortium has run online introductory courses on WGS in AMR surveillance; the WGS workflow—isolate to analysis; SARS-CoV-2 WGS; and basic bioinformatics using the Command line. The consortium has also scheduled in-person courses for a subset of >100 online trainees in Ghana who completed online courses on WGS sequencing and advanced bioinformatics using the Command line in September/October 2022 (https://antimicrobialresistance.dk/seqafrica/seqafrica-courses.aspx). In addition to the courses, coordinated sequencing at SEQAFRICA nodes has facilitated staff working at the centers, who received in-person training at DTU at the start of the project, to gain additional experience. Specifically, public health institutes that acquired Illumina and/or Nanopore capacity during the COVID-19 pandemic [11] can now apply these tools to bacterial genome sequencing. Consequently, the potential for SEQAFRICA and complementary initiatives, such as the Africa Pathogen Genomics Initiative, to support *S.* Typhi surveillance is substantial.

## TYPHI PATHOGENWATCH

Typhi Pathogenwatch [12] is a web application developed to support *S.* Typhi surveillance. The interface supports genome analytics, such as genotyping, detecting AMR determinants and plasmid replicons, and contextualization with genomic data. Typhi Pathogenwatch uses genome assemblies to perform 3 essential tasks for surveillance and epidemiological investigations: (1) placing isolates into lineages or clonal groups based on their genetic distance; (2) identifying their closest relatives and linking to their geographic distribution; and (3) detecting the presence of genes and mutations associated with AMR. In addition, Typhi Pathogenwatch provides compatibility with typing information for multilocus sequence typing (MLST), core genome MLST, in silico serotyping, the GenoTyphi genotyping scheme, and plasmid replicon sequences (PlasmidFinder).

**Figure 2.**
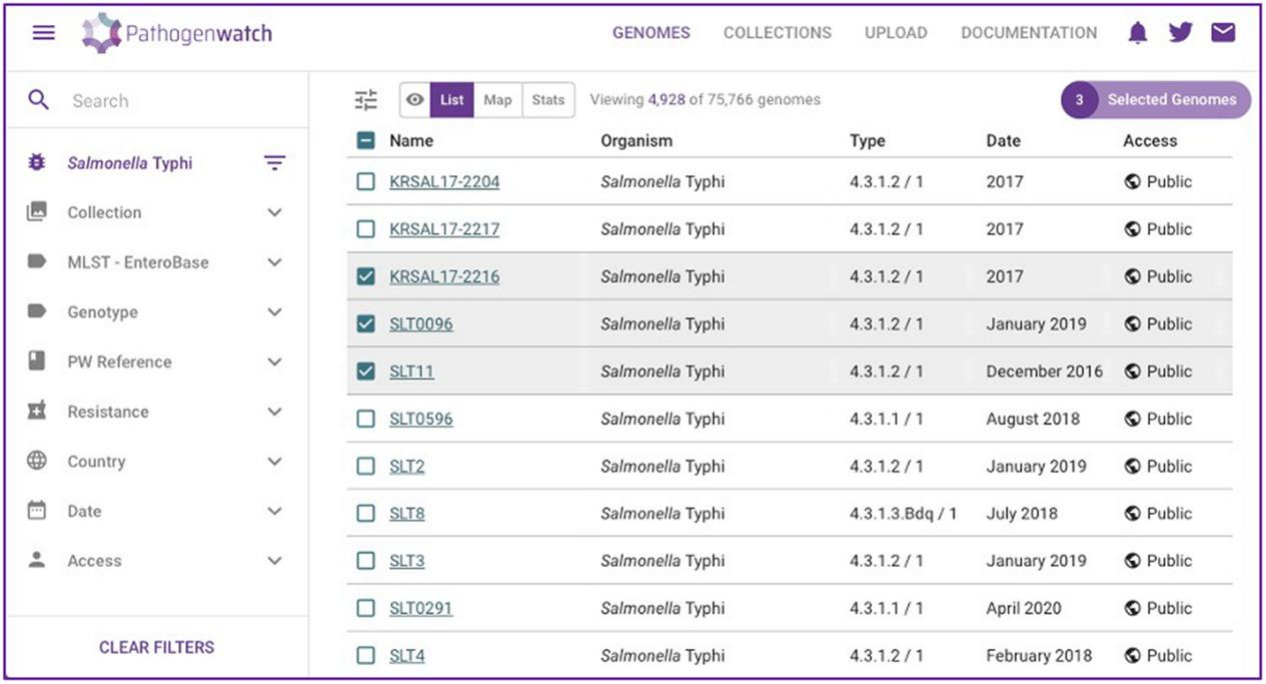
The Genomes page in Pathogenwatch allows the user to select a custom dataset of genomes based on available filters (left) or by clicking on individual genomes (ticked boxes). A collection can be created from selected genomes and all analytics can be downloaded.

The application can be accessed at https://pathogen.watch/styphi, where users can create an account to upload and analyze their genomes. User data remain private and stored in their personal account. Public genomes (N = 12 014 at the time of writing) available in Pathogenwatch with linked metadata are curated by the Global Typhoid Genomics Consortium. Metadata include, when available, country, date and source of isolation, travel information, patient age, and purpose of sampling. Users can browse and create custom collections of private and/or public genomes available in the application (https://pathogen.watch/genomes/all?organismId=90370) via a set of filters including country, date, MLST, genotype, and AMR (Figure 2).

**Figure 3.**
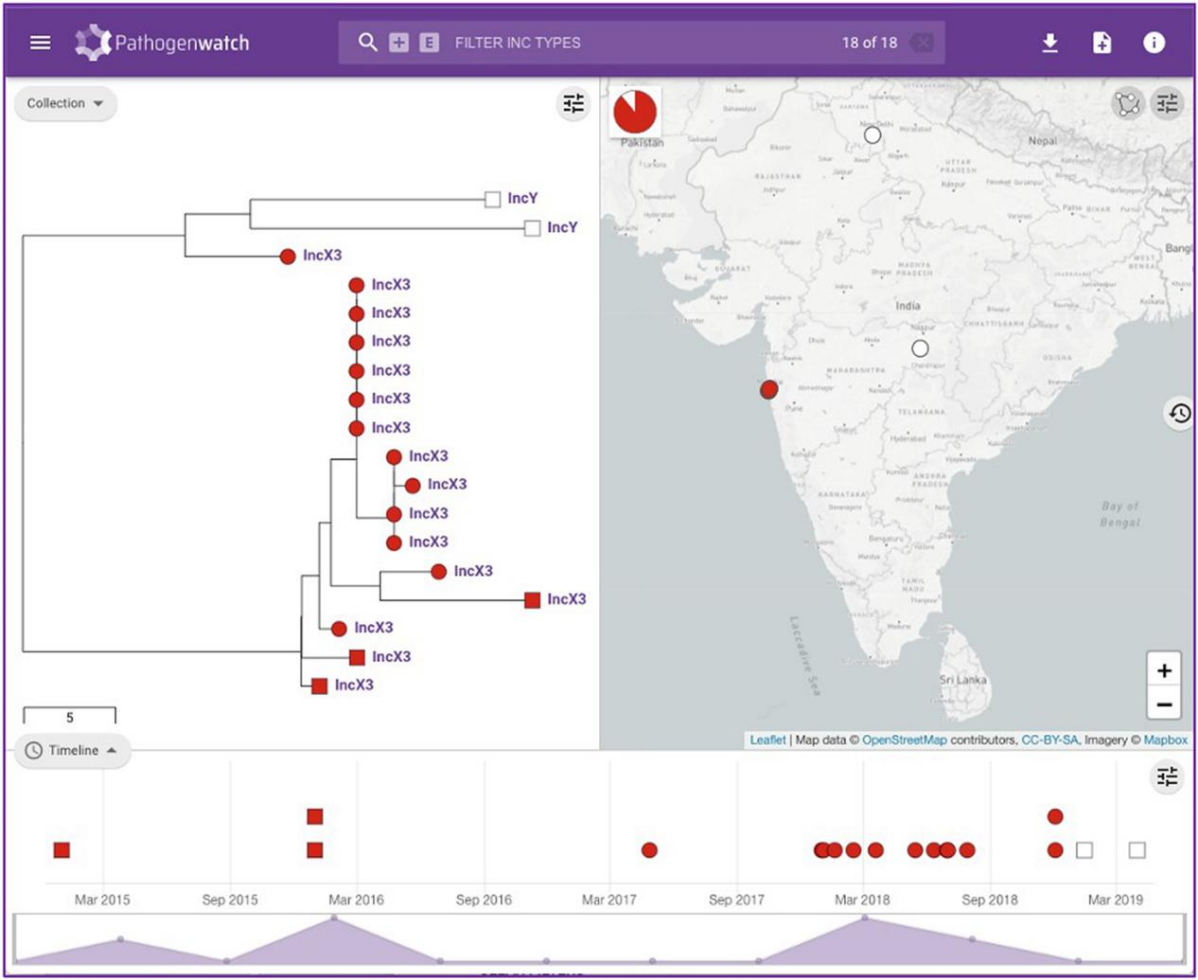
A collection of 18 genomes uploaded by the user (circles) or public (squares), selected from the Genomes page as resistant to broad-spectrum cephalosporins and from India, highlights the link between the *bla_SHV-12_* gene (red) and the IncX3 plasmid replicon sequence, and the *bla_CTX-M-15_* gene (white) and the IncY plasmid replicon.

The results for a single genome are displayed in a genome report that can be downloaded as a PDF. The results for a collection of genomes can be viewed online (Figure 3) and downloaded as trees and tables of genotypes, AMR predictions, assembly metrics, and typing information. Results can be accessed at a later date and shared via a collection identification number embedded in a unique weblink, thus facilitating international collaboration. An example of the output is shown in Figure 3: Resistance to third-spectrum cephalosporins in a subset of genomes from India could be explained by the presence of extended-spectrum β-lactamase genes, either *bla*_SHV-12_ in an IncX3 plasmid or *bla*_CTX-M-15_ in an IncY plasmid. Genomes with *bla*_SHV-12_ show a narrow geographic distribution (map) but wider temporal distribution (timeline), whereas genomes with *bla*_CTX-M-15_ show a wider geographic distribution but narrower temporal distribution.

Typhi Pathogenwatch combines accurate genomic predictions of AMR with broad geographic and population context within an easy-to-use interface accessible to users of all bioinformatics skills levels. Global genotype and AMR data generated by Typhi Pathogenwatch are utilized and aggregated by the TyphiNET dashboard. This approach allows the rapid and incremental addition of new data and can be used to underpin surveillance of typhoid and public health decision making at the local, national, and international scales.

## TYPHINET: AN ONLINE AMR SURVEILLANCE DASHBOARD FOR GLOBAL GENOMIC SURVEILLANCE OF *S.* TYPHI

TyphiNET is a recently developed online resource that aims to provide easy access to genome-derived data on the global distribution of *S.* Typhi genotypes and AMR determinants (http://typhi.net) [13]. TyphiNET empowers users to explore global trends in genome-derived metrics of public health utility, including AMR and genotype frequencies summarized down to national annual prevalence levels, without specialist computing technologies or bioinformatics expertise. Genotype and AMR data are imported directly from Typhi Pathogenwatch and filtered to include only genome collections representing nontargeted sampling, suitable for estimating national annual prevalence data. Input data can be further filtered to include only specified time periods, or to exclude data derived from returning travelers. Users can generate current reports and data visualizations of typhoid populations at a global and/or country level via any web browser.

**Figure 4.**
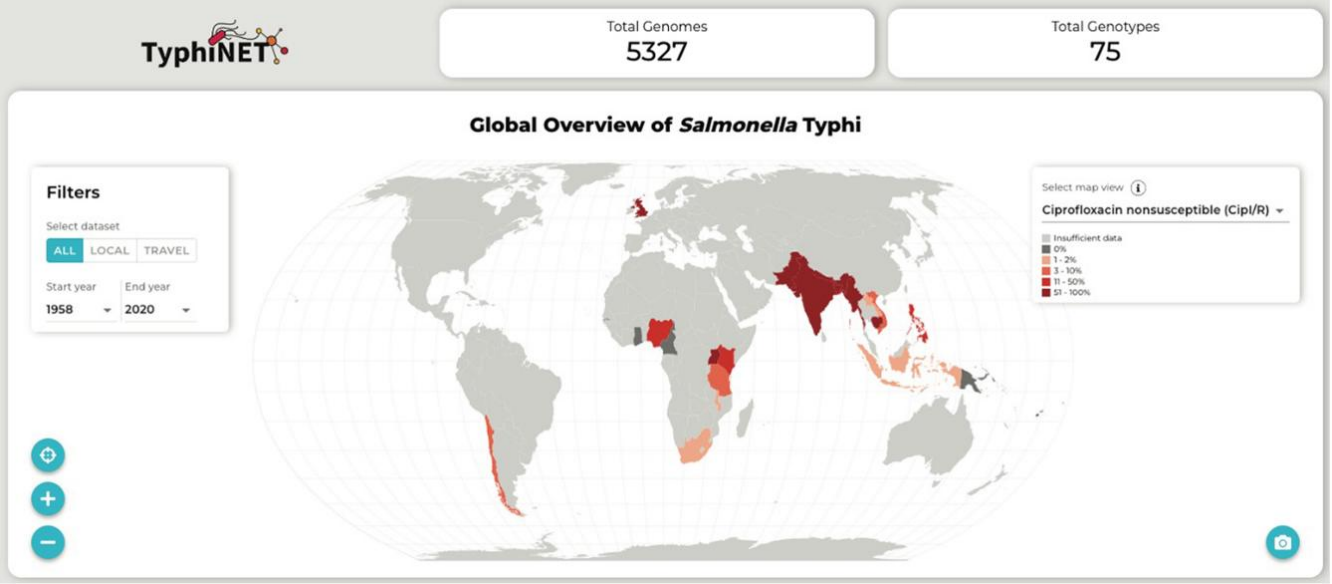
TyphiNET user-generated global overview of ciprofloxacin nonsusceptibility frequencies. Top panels indicate the number of sequences and genotypes present in the TyphiNET database as of May 2022. Left panel indicates controls for filtering the data visualized by data source (all data, locally collected, or travel-associated cases) and time period (by providing start and end years for the period). Countries on the map are colored by the frequency of ciprofloxacin nonsusceptibility as per the inset legend (top right of map). Data for each country are shown when ≥20 sequences are available. Controls to zoom in and out of the map and to center the map are available at the bottom left of the map. Hovering the mouse over any of the countries on the map provides summary statistics. Clicking on individual countries triggers country-level summaries (shown in Figure 5). The camera button allows users to download visualizations.

Global patterns of national genotype and AMR frequencies can be visualized on a world map (Figure 4), with countries colored to indicate prevalence ranges (estimated from genome data) for clinically relevant AMR phenotypes. Categories of AMR that can be visualized in this way are MDR (resistant to the classical first-line drugs chloramphenicol, co-trimoxazole, and ampicillin), azithromycin resistant, ciprofloxacin nonsusceptible, ciprofloxacin resistant, XDR (MDR plus ceftriaxone and ciprofloxacin resistant, ie, resistant to all oral drugs except for azithromycin), or susceptible to all relevant antimicrobials. Users can also generate maps showing the prevalence of genotype 4.3.1 (H58; commonly associated with AMR), the dominant genotype per country, and the number of genomes available for each country.

**Figure 5.**
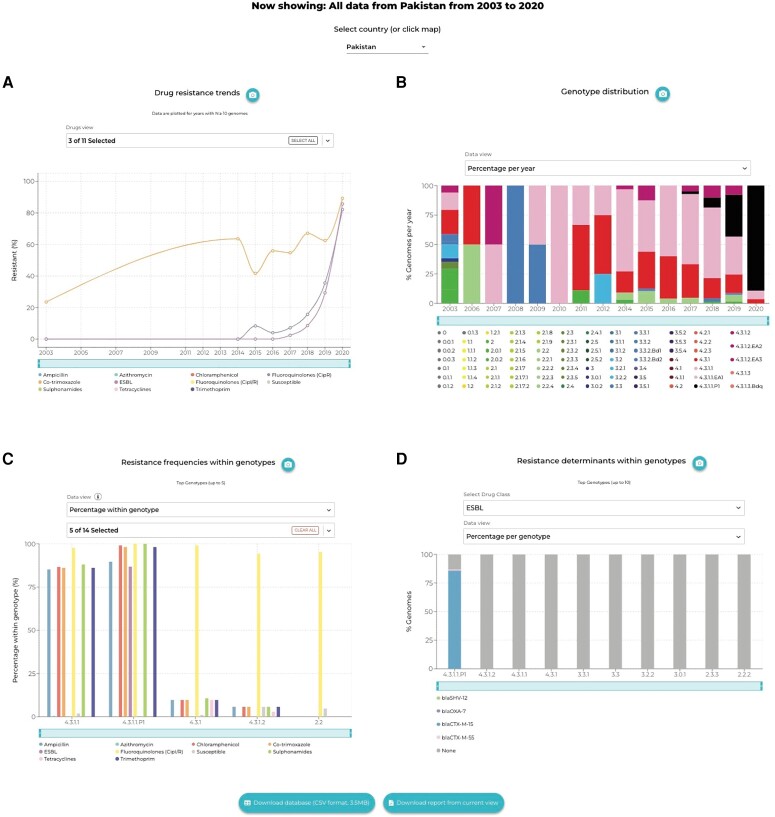
TyphiNET country-level overviews of *Salmonella* Typhi populations in Pakistan. *A*, Drug resistance trends over time plot shows frequencies of resistance to selected drug classes per year where ≥10 sequences are available. Lines are colored as per the inset legend; here we have selected “ESBL” (ie, third-generation cephalosporin [3GC] resistance, purple), ciprofloxacin resistance (blue/gray), and co-trimoxazole resistance (orange) to simplify the view. *B*, Annual genotype distribution plot reveals the frequencies of pathogen genotypes per year. Genotypes are colored as per the inset legend. *C*, Resistance frequencies within genotype plot shows frequencies of resistance to different drug classes, within common genotypes. Bars are colored according to the inset legend. The 5 most resistant genotypes are shown by default. *D*, Resistance determinants within genotype plot shows the distribution of specific genes and mutations mediating resistance to a selected drug class, within common pathogen genotypes. Bars are colored as per the inset legend. Buttons at the bottom of the page allow users to download a PDF report of the current plot views, as well as a line list of all genome-derived data in plain-text comma-separated variable (CSV) format. The camera button allows users to download visualizations.

The following plots can be viewed for either global data or a selected country (Figure 5): (1) AMR frequencies over time; (2) trends in genotype frequency over time; (3) the frequency of resistance to different drug classes among pathogen genotypes; and (4) the genes associated with AMR within pathogen genotypes. Country-level visualizations for Pakistan, using all available genomes from 2003–2020 including returning travelers, are shown in Figure 5 and illustrate the previously identified emergence and expansion of genotype 4.3.1.1.P1, responsible for the ongoing XDR outbreak in Pakistan [14, 15]. TyphiNET visualizations show an increase in the proportion of *S.* Typhi that are resistant to classical first-line drugs, ciprofloxacin, and third-generation cephalosporins (3GC) (Figure 5*A* ) from around 2017, coinciding with an increase in genotype 4.3.1.1.P1 (Figure 5*B* ). Examination of resistance frequencies within genotypes demonstrates that 3GC resistance is associated only with genotype 4.3.1.1.P1 (Figure 5*C* ) and mediated by a *bla_CTX-M-15_* gene (Figure 5*D* ).

TyphiNET provides easy access to aggregated genome-derived resistance frequencies for clinically relevant antimicrobials utilized in controlling typhoid, making these data accessible for the first time to a broad range of users without genomics expertise. It is anticipated that these data will be of public health utility as they have the potential to inform control strategies. For example, overviews of resistance frequencies could assist in guiding empirical treatment of typhoid in LMIC settings where the disease is likely endemic but surveillance data are lacking. In high-income countries where most infections are travel-associated, individual treatment could be informed by resistance frequencies from the country or countries visited. High resistance frequencies to multiple antimicrobials may be informative for targeting other intervention strategies such as the programmatic use of typhoid conjugate vaccines in specific regions. The inclusion of data from travel-associated cases provides informal sentinel surveillance for resistance in countries in which *S.* Typhi surveillance data are not available [16].

TyphiNET was developed as an open-source MERN (MongoDB, Express, React, Node.js) stack JavaScript application (code available at: https://github.com/zadyson/TyphiNET). Source, genotypes [17–19], and AMR data from “nontargeted” sampling frames sequences are imported regularly from Typhi Pathogenwatch [12] and curated by contributors to the Global Typhoid Genomics Consortium.

## GLOBAL TYPHOID GENOMICS CONSORTIUM

The Global Typhoid Genomics Consortium was established in April 2021 to provide a mechanism for the global typhoid research community to engage in the aggregation of *S.* Typhi genomic data, to facilitate monitoring the emergence and spread of AMR and to inform targeted public health action. The specific goals of the consortium are to (1) encourage prompt sharing of typhoid genome data for public health benefit; (2) facilitate the extraction and reporting of key data of public health relevance; and (3) promote and facilitate the dissemination and use of information derived from typhoid genomic data to monitor AMR and postvaccination impact.

The key activities of the consortium are to encourage and coordinate sharing and release of typhoid genomics data in a manner that maximizes its potential to inform public health. The consortium does not seek to generate or claim ownership of any genome data; rather, the model is to encourage data generators to deposit raw genome data into public databases and share source information using a standardized metadata template, available at https://bit.ly/typhiMeta. This model facilitates harmonization of source information across aggregated *S.* Typhi data, which is crucial to allow downstream integration of the data for public health benefit, while ensuring that consortium members retain full control over their data and when they choose to make it public. Typhi Pathogenwatch is used as the central analysis platform to generate inferred genotypes and AMR determinants from raw genomes, and to maintain a publicly available and searchable database of genome assemblies and an interactive global phylogeny. Genotyping is conducted using the GenoTyphi genotyping framework [17, 18], whose ongoing curation will be managed by a working group of the consortium. A key field in the consortium metadata template is “purpose of sampling,” which seeks to identify sets of genomes that are derived from “nontargeted” sampling frames that are suitable for estimation of national annual prevalence rates of AMR and genotypes (eg, in the TyphiNET dashboard, described above, and other reports).

Consortium membership is free and open to all (https://www.typhoidgenomics.org/); the intention is to include all those with an academic or public health interest in using WGS to investigate, monitor, and/or understand typhoid epidemiology. Current membership (as at mid-2022) numbers >150 individuals from 39 countries. The majority of members are from countries in Africa and Asia where typhoid is endemic; however, there is also considerable participation from countries where typhoid is considered a travel-associated disease and subject to routine WGS, which provides useful “sentinel surveillance” data for common travel destinations [16]. Consortium activities are overseen by a multidisciplinary steering committee of international experts in typhoid surveillance and epidemiology, and an advisory board of stakeholders from the global public health community has been engaged to help identify ways to promote typhoid genomic surveillance—and particularly the use of pathogen WGS data—for public health benefit.

## CONCLUSIONS AND NEXT STEPS

The global response to the SARS-CoV-2 pandemic has demonstrated the value of global genomic surveillance and timely data sharing in monitoring the emergence and spread of pathogenic strains, and one of its legacies will be broader access to and use of sequencing and phylogenetic analysis [20]. Such capacity could be expanded to include surveillance of *S.* Typhi and other priority pathogens if this is not already being done, and *S.* Typhi could be incorporated into genomic initiatives conducted at the local, national, and regional levels. Generating additional, more geographically representative *S.* Typhi WGS data and standardized metadata and sharing these data more broadly can facilitate a better understanding of the global distribution of drug-resistant *S.* Typhi, as well as how and where AMR emerges and spreads, thereby providing additional incentives for timely, open data sharing. By sharing information about platforms and programs that have been established to enable the generation, analysis, and visualization of *S.* Typhi genomic data, both on the African continent and more broadly, we hope to encourage further uptake of these freely available resources, which may lead to additional, sustainable generation of *S.* Typhi genomic data to inform decision making. In addition, we hope that governments, donors, and other stakeholders continue to support the establishment and sustenance of molecular surveillance capacity by providing funding for procurement of equipment and reagents, training for bioinformaticians, establishment of quality-assured laboratories, and sample management.

## Notes

***Acknowledgments.*** I. N. O. and A. M. S. are grateful to the SEQAFRICA lead collaborators who conceptualized and run the program, in particular Rene Hendriksen, Beverly Egyir, Marco van Zwetselaar, and Pernille Nilsson.

***Financial support.*** SEQAFRICA is a regional grant funded by the Department of Health and Social Care and managed by the Fleming Fund. The Fleming Fund is a £265 million UK aid program supporting up to 24 low- and middle-income countries that generate, share, and use data on antimicrobial resistance and works in partnership with Mott MacDonald, the management agent for the regional grants program. I. N. O. is a Calestous Juma Science Leadership Fellow supported by the Bill & Melinda Gates Foundation. Pathogenwatch is developed with support from the Li Ka Shing Foundation (Big Data Institute, University of Oxford) and Wellcome (grant number 099202). The TyphiNET project has received funding from the Wellcome Trust (Open Research Fund, 219692/Z/19/Z awarded to K. E. H.) and the European Union’s Horizon 2020 research and innovation program under the Marie Sklodowska-Curie grant agreement number 845681 (awarded to Z. A. D.). K. E. H. was supported by a Senior Medical Research Fellowship from the Viertel Foundation of Australia, and by the Bill & Melinda Gates Foundation (grant number OPP1175797).

***Supplement sponsorship.*** This article appears as part of the supplement “Charting the Course to Meet the Challenges Ahead: Research and Developments on Typhoid and Other Invasive Salmonelloses” sponsored by the Coalition against Typhoid Secretariat, housed at the Sabin Vaccine Institute in Washington, DC and made possible by a grant from the Bill & Melinda Gates Foundation.

## References

[1] Yousafzai MT , Karim S, Qureshi S, et al Effectiveness of typhoid conjugate vaccine against culture-confirmed *Salmonella enterica* serotype Typhi in an extensively drug-resistant outbreak setting of Hyderabad, Pakistan: a cohort study. Lancet Glob Health 2021; 9:e1154–62. 34297962 10.1016/S2214-109X(21)00255-2 PMC8315145

[2] Okeke IN , Feasey N, Parkhill J, et al Leapfrogging laboratories: the promise and pitfalls of high-tech solutions for antimicrobial resistance surveillance in low-income settings. BMJ Glob Health 2020; 5:e003622. 10.1136/bmjgh-2020-003622 PMC7712442 33268385

[3] Africa Centres for Disease Control and Prevention . Institute of Pathogen Genomics (IPG). 2022. Available at: https://africacdc.org/institutes/ipg/. Accessed 3 February 2023.

[4] Inzaule SC , Tessema SK, Kebede Y, Ogwell Ouma AE, Nkengasong JN. Genomic-informed pathogen surveillance in Africa: opportunities and challenges. Lancet Infect Dis 2021; 21:e281–9. 33587898 10.1016/S1473-3099(20)30939-7 PMC7906676

[5] Ikhimiukor OO , Odih EE, Donado-Godoy P, Okeke IN. A bottom-up view of antimicrobial resistance transmission in developing countries. Nat Microbiol 2022; 7:757–65. 35637328 10.1038/s41564-022-01124-w

[6] von Kalckreuth V , Konings F, Aaby P, et al The Typhoid Fever Surveillance in Africa Program (TSAP): clinical, diagnostic, and epidemiological methodologies. Clin Infect Dis 2016; 62:S9–16. 26933028 10.1093/cid/civ693 PMC4772831

[7] Makungo UB , Ramutshila TE, Mabotja MC, et al Epidemiological investigation of a typhoid fever outbreak in Sekhukhune District, Limpopo province, South Africa—2017. S Afr J Infect Dis 2020; 35:107. 34485467 10.4102/sajid.v35i1.107 PMC8378196

[8] Smith AM , Tau NP, Ngomane HM, et al Whole-genome sequencing to investigate two concurrent outbreaks of *Salmonella enteritidis* in South Africa 2018. J Med Microbiol 2020; 69:1303–7. 33048044 10.1099/jmm.0.001255

[9] Ikhimiukor OO , Oaikhena AO, Afolayan AO, et al Genomic characterization of invasive typhoidal and non-typhoidal *Salmonella* in southwestern Nigeria. PLoS Negl Trop Dis 2022;16:e0010716. 10.1371/journal.pntd.0010716 PMC9455843 36026470

[10] Yamba K , Kapesa C, Mpabalwani E, et al Antimicrobial susceptibility and genomic profiling of *Salmonella enterica* from bloodstream infections at a tertiary referral hospital in Lusaka, Zambia, 2018–2019. IJID Regions 2022; 3:248–55. 35755477 10.1016/j.ijregi.2022.04.003 PMC9216281

[11] Wilkinson E , Giovanetti M, Tegally H, et al A year of genomic surveillance reveals how the SARS-CoV-2 pandemic unfolded in Africa. Science 2021; 374:423–31. 34672751 10.1126/science.abj4336 PMC7613315

[12] Argimón S , Yeats CA, Goater RJ, et al A global resource for genomic predictions of antimicrobial resistance and surveillance of *Salmonella* Typhi at Pathogenwatch. Nat Commun 2021; 12:2879. 34001879 10.1038/s41467-021-23091-2 PMC8128892

[13] Dyson ZA , Cerdeira L. zadyson/TyphiNET: v1.2.1. Zenodo, 2022. 10.5281/zenodo.5338903

[14] Klemm EJ , Shakoor S, Page AJ, et al Emergence of an extensively drug-resistant *Salmonella enterica* serovar typhi clone harboring a promiscuous plasmid encoding resistance to fluoroquinolones and third-generation cephalosporins. mBio 2018; 9:1–10. 10.1128/mBio.00105-18 PMC5821095 29463654

[15] Rasheed MK , Hasan SS, Babar ZUD, Ahmed SI. Extensively drug-resistant typhoid fever in Pakistan. Lancet Infect Dis 2019; 19:242–3. 30833059 10.1016/S1473-3099(19)30051-9

[16] Ingle DJ , Nair S, Hartman H, et al Informal genomic surveillance of regional distribution of *Salmonella* Typhi genotypes and antimicrobial resistance via returning travellers. PLoS Negl Trop Dis 2019; 13:1–20. 10.1371/journal.pntd.0007620 PMC6741848 31513580

[17] Dyson ZA , Holt KE. Five years of GenoTyphi: updates to the global *Salmonella* Typhi genotyping framework. J Infect Dis 2021; 224:775–80. 10.1093/infdis/jiab414 PMC8687072 34453548

[18] Wong VK , Baker S, Connor TR, et al An extended genotyping framework for *Salmonella enterica* serovar Typhi, the cause of human typhoid. Nat Commun 2016; 7:1–11. 10.1038/ncomms12827 PMC5059462 27703135

[19] Dyson ZA , Holt K. katholt/genotyphi: GenoTyphi v1.9.1. Zenodo, 2021. 10.5281/zenodo.4707614.

[20] Belman S , Saha S, Beale MA. SARS-CoV-2 genomics as a springboard for future disease mitigation in LMICs. Nat Rev Microbiol 2022; 20:3. 34799703 10.1038/s41579-021-00664-y PMC8603645

